# Chronic Intrinsic Transient Tracheal Occlusion Elicits Diaphragmatic Muscle Fiber Remodeling in Conscious Rodents

**DOI:** 10.1371/journal.pone.0049264

**Published:** 2012-11-01

**Authors:** Barbara K. Smith, A. Daniel Martin, Krista Vandenborne, Brittany D. Darragh, Paul W. Davenport

**Affiliations:** 1 Department of Physical Therapy, College of Public Health and Health Professions, University of Florida, Gainesville, Florida, United States of America; 2 College of Medicine, University of South Florida, Tampa, Florida, United States of America; 3 Department of Physiological Sciences, College of Veterinary Medicine, University of Florida, Gainesville, Florida, United States of America; Universidad Europea de Madrid, Spain

## Abstract

**Background:**

Although the prevalence of inspiratory muscle strength training has increased in clinical medicine, its effect on diaphragm fiber remodeling is not well-understood and no relevant animal respiratory muscle strength training-rehabilitation experimental models exist. We tested the postulate that intrinsic transient tracheal occlusion (ITTO) conditioning in conscious animals would provide a novel experimental model of respiratory muscle strength training, and used significant increases in diaphragmatic fiber cross-sectional area (CSA) as the primary outcome measure. We hypothesized that ITTO would increase costal diaphragm fiber CSA and further hypothesized a greater duration and magnitude of occlusions would amplify remodeling.

**Methodology/Principal Findings:**

Sprague-Dawley rats underwent surgical placement of a tracheal cuff and were randomly assigned to receive daily either 10-minute sessions of ITTO, extended-duration, 20-minute ITTO (ITTO-20), partial obstruction with 50% of cuff inflation pressure (ITTO-PAR) or observation (SHAM) over two weeks. After the interventions, fiber morphology, myosin heavy chain composition and CSA were examined in the crural and ventral, medial, and dorsal costal regions. In the medial costal diaphragm, with ITTO, type IIx/b fibers were 26% larger in the medial costal diaphragm (p<0.01) and 24% larger in the crural diaphragm (p<0.05). No significant changes in fiber composition or morphology were detected. ITTO-20 sessions also yielded significant increases in medial costal fiber cross-sectional area, but the effects were not greater than those elicited by 10-minute sessions. On the other hand, ITTO-PAR resulted in partial airway obstruction and did not generate fiber hypertrophy.

**Conclusions/Significance:**

The results suggest that the magnitude of the load was more influential in altering fiber cross-sectional area than extended-duration conditioning sessions. The results also indicated that ITTO was associated with type II fiber hypertrophy in the medial costal region of the diaphragm and may be an advantageous experimental model of clinical respiratory muscle strength training.

## Introduction

The diaphragm is the primary muscle of the inspiratory pump, and in humans it contracts every three to five seconds to sustain alveolar ventilation. Because diaphragmatic contractile activity is vital, it is important to understand how the muscle responds to changes in motor activity or environmental conditions. For example, controlled mechanical ventilation (MV) weakens the inspiratory pump and results in significant diaphragmatic oxidative stress, proteolysis and atrophy after as little as six hours in small mammals [1]–[3]. The ventilated human diaphragm can also atrophy and lose contractile function rapidly, within three to seven days of controlled MV [4]–[6]. In contrast, inspiratory muscle strength training (IMST) with high pressure threshold loads has been shown to increase the pressure generating capacity of the inspiratory pump and facilitate weaning from MV in hospitalized patients [7]–[10]. Since more than half of unweaned patients do not survive one year [11], [12] and the annual medical costs are an estimated $3.5 million/survivor [12], it is important to identify treatments that can prevent or reverse ventilator-induced diaphragm dysfunction. However, the effects of IMST on muscular remodeling are virtually unknown for the human diaphragm, because the location of the muscle makes it problematic to access for biopsy. Therefore, an animal model of IMST would enable systematic investigations on the histological, molecular, and functional plasticity of training on the respiratory muscles.

Previous animal studies reveal that mechanical overloads can remodel diaphragmatic fibers according to the overload type, intensity, and duration. Sustained resistive loading by tracheal banding causes hypertrophy of slow muscle fibers in hamsters [13] and rats [14] but is also associated with high mortality. Shorter yet physiologically challenging durations of resistive breathing increase diaphragmatic slow MHC gene expression, with concurrent plasma membrane damage and sarcomere disruption [15], [16]. These studies suggested that the high magnitude and long duration of the loads damaged diaphragmatic fibers. Indeed, morphological evidence of human diaphragm injury has been reported following prolonged, fatiguing overloads [17]. Notably, none of the previous animal studies utilized intense, yet brief pressure loading regimes that resembled a clinical IMST exercise prescription in intensity and duration.

**Figure 1:**
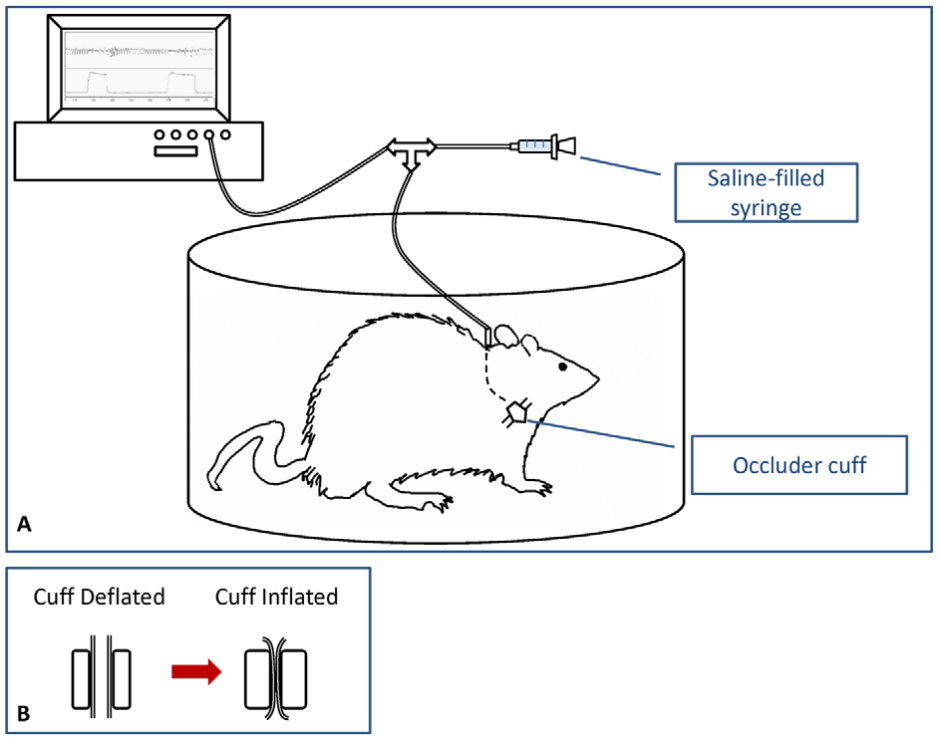
Illustration of ITTO experimental setup. (**A**) ITTO was administered in a plethysmograph to permit limited animal movement. The tracheal cuff was inflated by injection of sterile saline into the actuating line. The timing and magnitude of cuff pressure was monitored with a polygraph. (**B**) Cut-out illustration of the tracheal cuff. The trachea was fully patent with the cuff deflated. With cuff inflated, the trachea was fully and reversibly occluded.

Although the diaphragm comprises ∼0.5% of body mass (37), it spans a large relative surface area, and its muscular attachments to the thoracic cage may yield regional biomechanical differences. Differences in rodent costal and crural regions have been noted for myosin heavy chain (MHC) composition and metabolic responses to whole-body endurance exercise [18]–[20]. The magnitude of load-induced remodeling also appears to be greater in the costal regions [21]. Within the costal diaphragm, local differences in the zone of apposition surface area and muscle thickness are thought to influence the local pleural pressure and shortening [22]. Regional variations in rodent diaphragm thickness [23] and in blood flow during exercise [24] suggests that muscular load compensations could vary locally in response to activity. However, the regional histological remodeling of the rodent diaphragm has not been systematically explored with strength training.

**Table 1:** Demographic information in sham-occluded animals and animals treated with ITTO.

	ITTO	ITTO-20	ITTO-PAR	SHAM
*Age (weeks)* *				
Study onset	8.9 (8.6–9.5)	19.7 (10.9–36.5) ^‡^	12.4 (12.3–12.7) ^‡^	9.6 (9.3–17.4)
*Body mass (g*)^ †^				
Study onset	262±45	330±76 §	346±14	273±46
Study conclusion	304±19	347±72 §	362±24	291±31

* Values are median (IQR). ^†^ Values are mean ± SD. ^‡^ Significant difference from ITTO, p<0.05. § Significant difference from ITTO, p<0.01.

In humans, IMST uses a load characterized by an inspiratory occlusion until the inspiratory muscles generate sufficient pressure to open a pre-set pressure threshold valve. We employed a rodent model of intrinsic transient tracheal occlusion (ITTO) to provide brief, reversible occlusive loads to respiration. The principal objective was to investigate whether this novel ITTO loading paradigm could elicit diaphragmatic fiber hypertrophy without inducing the fiber injury reported with sustained loading such as tracheal banding. This project was based on the underlying postulate that short duration, high intensity ITTO would elicit diaphragmatic fiber remodeling, according to both the imposed training regime as well as the regional mechanical efficiency of the loaded muscle. Our findings indicate ITTO could be a useful experimental model of the respiratory muscle hypertrophy induced by the initial occlusion-load phase of IMST.

**Figure 2:**
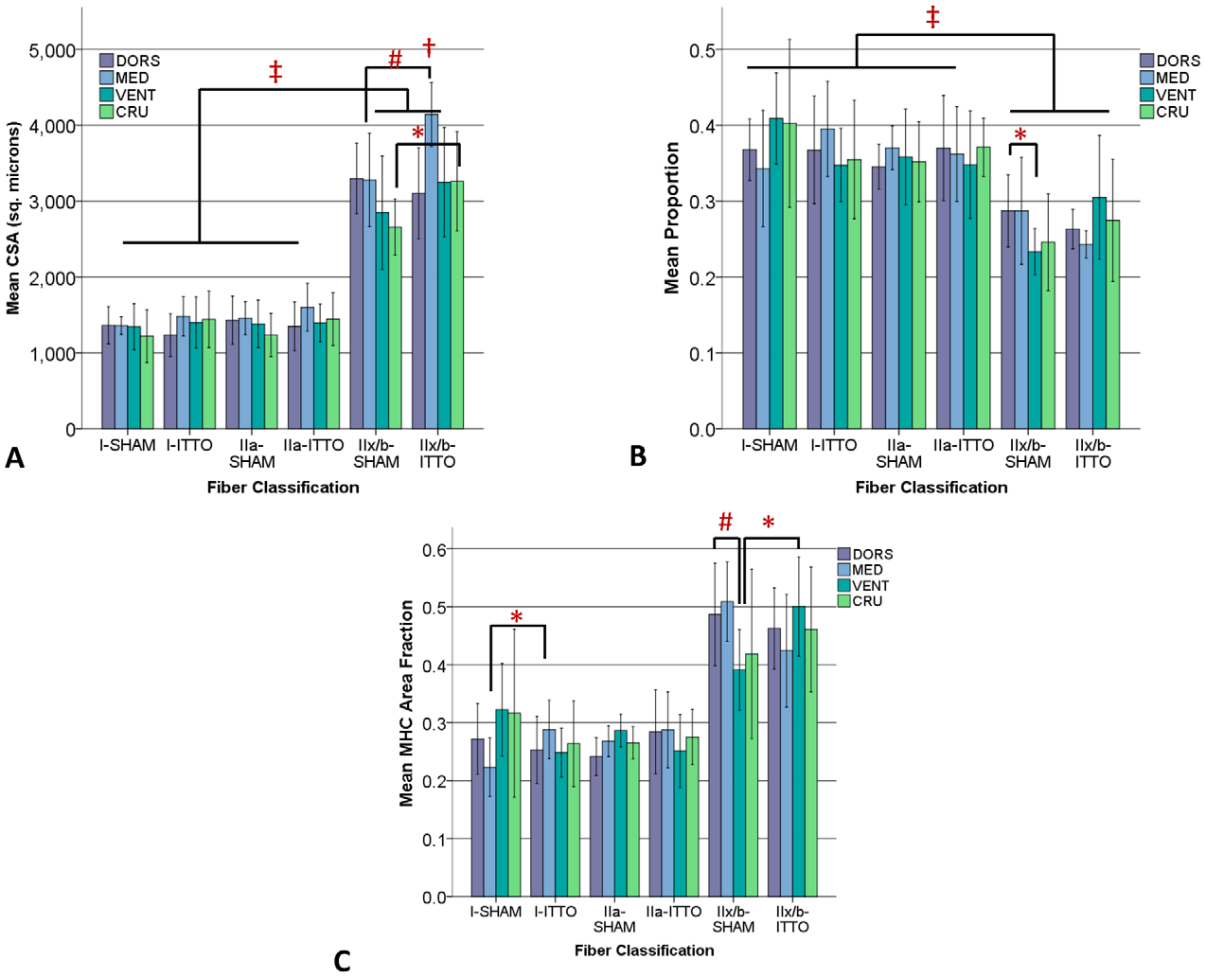
Regional remodeling of the diaphragm, following ITTO or SHAM training. (**A**) **CSA.** Type IIx/b CSA in the medial costal (#) and crural (*) regions was significantly greater in ITTO animals than SHAM animals. In the ITTO group, type IIx/b medial costal diaphragm fibers were significantly larger than dorsal, ventral and crural segments (†). In both groups, type IIx/b fibers were significantly larger than oxidative fibers (‡). (**B**) **Fiber proportions.** In the SHAM group, the ventral region contained significantly fewer type IIx/b fibers than the dorsal region (*). In all groups, type IIx/b fibers were significantly less prevalent than type IIa or I fibers (‡). (**C**) **MHC A_A_.** The type I fiber A_A_ of the medial region was significantly larger in ITTO animals than SHAM animals (*). The A_A_ of type IIx/b fibers of the SHAM ventral diaphragm was significantly lower than the SHAM medial diaphragm (^#^) and the ITTO ventral diaphragm (*). (Error bars are ± 1SD, *p<0.05; ^#^ p<0.01; ‡ p<0.001; † p<0.05 vs other regions).

In characterizing the diaphragmatic fiber remodeling produced by this innovative mode of training, three specific aims were addressed: (i) identification of regional remodeling differences, (ii) examination of the effect of training session duration on remodeling, and (iii) a comparison of occlusive versus resistive overloads. We hypothesized that ITTO would produce fast-fiber hypertrophy in the costal diaphragm, compared to a sham-trained control group. We further hypothesized that increasing the duration of daily training sessions increases this hypertrophy. Finally, we hypothesized that partial airway obstruction would not cause fiber hypertrophy.

**Figure 3:**
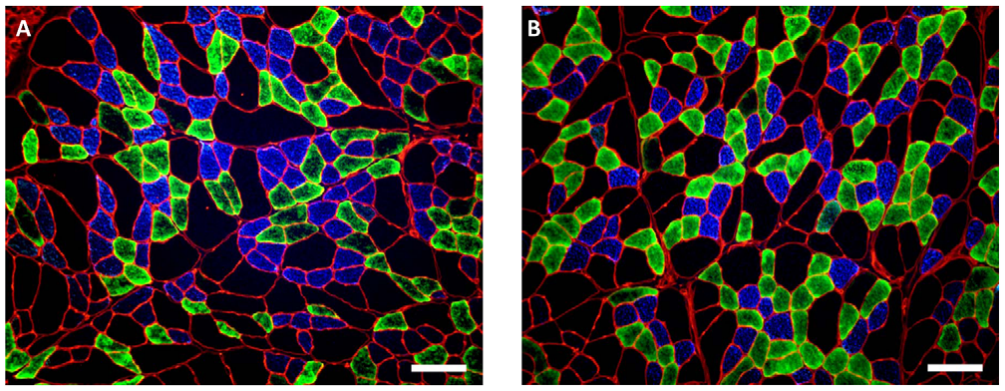
Immunofluorescent analysis of myosin heavy chain in the medial costal diaphragm. (**A**) Diaphragm from ITTO animal. (**B**) Diaphragm from SHAM animal. Type I fibers fluoresced blue, type IIa illuminated green, and type IIx/b fibers remained free of fluorescence. Images were captured with 100X magnification. Scale bar represents 100 µm.

## Materials and Methods

### Ethical Approval

The study was carried out in accordance with the procedures outlined in the Guide for the Care and Use of Laboratory Animals of the National Institutes of Health. The study was approved by the University of Florida Institutional Animal Care and Use Committee (Project #E575).

**Table 2:** Area fraction (A_A_) of remodeled cells in the costal and crural diaphragm.

	Normal fibers %	Remodeled fibers* %	Connective tissue* %
*Dorsal costal*			
SHAM	83.3±1.9	4.8±0.9	9.0±0.7
ITTO	82.7±4.5	5.0±2.0	9.5±1.8
*Medial costal*			
SHAM	85.7±4.6	4.0±1.8	10.3±6.0
ITTO	85.5±2.4	3.7±1.6	10.8±2.8
*Ventral costal*			
SHAM	83.3±3.8	4.9±1.9	9.2±3.5
ITTO	80.3±9.4	6.6±4.9	10.3±3.8
*Crural*			
SHAM	83.5±3.3	3.9±1.6	12.6±2.1
ITTO	84.3±4.2	3.4±1.6	12.3±3.5

Values are mean ± SD. *p<0.001 Significant main effect for category: differences between A_A_ of normal, remodeled and connective tissue.

### Animals

Twenty-nine male Sprague-Dawley rats were studied (Harlan Laboratories, Indianapolis, IN). Animals were maintained in standard housing in the University of Florida animal care facility. A 12∶12 hour reverse light: dark cycle and *ad libitum* diet were provided to the animals throughout the experiments. The ITTO training regime was based on experiments designed by our laboratory to investigate the neurobiology of respiratory mechanosensation and motor compensation to occlusive loads [25]–[27]. All animals underwent placement of a tracheal cuff and were then randomly assigned to a conditioning group. The study aims were carried out in separate experiments: (1) the effect of ITTO on the regional muscle remodeling of the diaphragm, and (2) the effect of modified ITTO training regimes on remodeling of the medial costal diaphragm.

### Occluder Placement

During occluder placement, animals were anesthetized using isoflurane gas (2–5% in O_2_) and breathed room air spontaneously. A surgical plane of anesthesia was confirmed by absence of paw pinch and corneal reflexes, and then the trachea was exposed with a ventral incision. A vascular occluder (#18080-01, Fine Science Tools, Foster City, CA) was sutured around the trachea, and the actuating line was externalized dorsally between the scapulae. To inflate the occluder cuff bladder, the actuating line was connected to a saline-filled syringe. The inflated cuff closed the trachea and elicited ITTO. Deflation of the cuff fully restored airway patency and unobstructed breathing. The actuator line was stitched in place, and the tracheal incision sutured.

During the surgery and recovery, body temperature was maintained at 37°C with a heating pad. Animals received buprenorphine (.01–.05 mg/kg BW) and carprofen (5 mg/kg BW) for analgesia and were rehydrated (subcutaneous normal saline, .01–.02 mL/g BW) prior to withdrawal of anesthesia. Animals were closely monitored for signs of respiratory distress, infection, or pain. During a 4-day recovery, routine analgesics and anti-inflammatory medications (buprenorphine .01–.05 mg/kg BW every 12–24 hours and carprofen 5 mg/kg BW every 24 hours) were provided. This procedure is described in detail elsewhere [26].

### Training Protocol

After a 5–7-day recovery, animals underwent eleven sessions of the assigned training intervention. Sessions occurred in the morning and lasted up to 30 minutes. During session one, animals acclimatized to an observation chamber for ∼20 minutes. The tracheal cuff was not occluded. Figure 1 shows an illustration of the experimental setup and balloon cuff inflation.

#### Sham Training Group

In subsequent daily sessions (days 2–11), animals in the sham-conditioned (SHAM) group (N = 9, age 9.6 (9.3–17.4) weeks) were placed in an observation chamber for 15 minutes. The SHAM animals received no other interventions during observation sessions.

#### ITTO Training

Animals in the ITTO group (N = 8, age 8.9 (8.6–9.5) weeks) completed training sessions of 2.5 minutes of unobstructed breathing, 10 minutes of ITTO, followed by 2.5 minutes of unobstructed breathing. In preliminary experiments on excised tracheas, a cuff pressure of 400-600 mm Hg reliably produced reversible, complete occlusion. During ITTO, the tracheal cuff was manually inflated for 5–8 seconds in order to elicit 5–10 strong respiratory attempts, and then deflated for 20–25 seconds (∼100–150 occluded breaths over a 10-minute session). Due to the rapid respiratory rate of the conscious rats, some cuff inflations likely occurred at other phases beyond end-exhalation. Analogue tracings of the cuff pressure confirmed the onset and removal of ITTO [27].

To determine whether extended-duration training sessions enhanced fiber remodeling, an additional group (N = 6, age 19.7 (10.9–36.5) weeks) of animals received 20-minute sessions of ITTO (ITTO-20). ITTO-20 sessions consisted of a 2.5-minute unobstructed acclimation, followed by 20 minutes of ITTO, and a 2.5-minute unobstructed recovery. During ITTO-20, the tracheal cuff was inflated for 3–5 seconds to elicit 4–6 respiratory attempts, and then deflated for 10–15 seconds. While the ITTO-20 inflation period was shorter, the rest period between occlusions was also reduced, resulting in ∼300–350 occluded breaths over 20 minutes.

To determine remodeling differences between resistive and occlusive loads, a final group (N = 6) underwent partial tracheal occlusion (ITTO-PAR). The cuff pressure was 50% of the pressure required to fully occlude the trachea (200–300 mm Hg). Animals underwent 3–5 seconds of ITTO-PAR with 10–15 seconds of unobstructed recovery, for 20 minutes (∼300–350 resistive breaths during 20 minutes). A patent airway was confirmed during ITTO-PAR on training days 5 and 10, using capnography, P_es_ and flow tracings.

### Tissue Harvest

One day after the last session, animals were anesthetized with isoflurane gas (2–5% in O_2_) to a surgical plane of anesthesia. Once an absence of corneal and paw pinch reflexes was confirmed, animals were euthanized by decapitation. To identify regional variations in diaphragm fiber remodeling, diaphragms were extracted from SHAM and ITTO animals and divided into crural and dorsal, medial, and ventral costal regions as illustrated by Poole [24]. In ITTO-20 and ITTO-PAR animals, medial costal diaphragm tissue was preserved. The right hemidiaphragm was equilibrated at 4°C for 3–5 minutes at resting length as described elsewhere [4], [28], [29], prepared with embedding medium and then quick-frozen in liquid nitrogen-chilled isopentane. Specimens were stored in a −80°C freezer until analysis.

### Immunofluorescent Analysis

Ten-micron transverse serial specimens were sectioned for histological processing (Microm HM505 cryostat, Walldorf, Germany). Slides were permeabilized with 0.5% Triton-X100 in phosphate-buffered saline (PBS). The samples were incubated with primary antibodies for laminin (1∶200, anti-rabbit, IgG, Lab Vision, Kalamazoo, MI), type I myosin heavy chain (1∶15, anti-mouse A4.840, IgM, Developmental Studies, Iowa City, IA), and type IIa myosin heavy chain (1∶50, anti-mouse SC-71, IgG, Developmental Studies), followed by treatment with secondary antibodies for rhodamine (1∶40, goat-anti-rabbit, Invitrogen, Grand Island, NY), Alexa Fluor 350 (1∶333, goat-anti-mouse, IgM, Invitrogen) and Alexa Fluor 488 (1∶133, goat-anti-mouse, IgG, Invitrogen). Cover slips were mounted with Vectashield fluorescent mounting medium (Vector Labs, Burlingame, CA) and secured with nitrocellulose lacquer.

Samples were visualized using fluorescence microscopy at 10x magnification and N21, GFP, and A4 cube filters (Leica DM LB, Solms, Germany). Fiber cross-sectional area (CSA) was calculated from encoded images, using at least 250 fibers per specimen, with Scion Image (NIH) software. An investigator blinded to group assignment quantified CSA, fiber type, and the area fraction (A_A_) occupied by each MHC isoform.

### Muscle Fiber Morphology

Fiber CSA was selected over whole muscle weight as the primary indicator of training, because it is a sensitive early marker of hypertrophy [29] and enabled the investigation of regional remodeling. For muscle fiber morphology, 10-µm transverse serial sections were obtained from a cryostat cooled to −20°C (Microm HM505, Walldorf, Germany). Sections were stained with hematoxylin and eosin, and specimens were visualized with brightfield microscopy (Leica DM LB, Solms Germany) at 40x magnification. Twenty randomly-selected digital images were analyzed from each diaphragm specimen.

Tissue morphology was analyzed using a systematic point-counting technique validated by others [21], [30] and Adobe Photoshop CS3 software (Adobe Corporation, San Jose CA). Tissue was classified into one of nine morphological categories as described by Reid [21]: remodeled tissue included small, angular fibers, centrally-nucleated fibers, cytoplasmic changes, inflamed/necrotic fibers, and inflammatory cells. Connective tissue included interstitial spaces, collagen, and adipose tissue. Area fractions (A_A_) of normal muscle, connective tissue, and remodeled tissue were calculated.

### Statistical Analysis

Statistical analysis was conducted using SigmaStat 3.5 software (Systat Software, Inc., Chicago, IL). Kolmogorov-Smirnov and Shapiro-Wilk tests were used to examine the normality and homogeneity of variance of the data. Group descriptors were characterized with Kruskal-Wallace ANOVA (age) and repeated-measures ANOVA (body mass). Analysis of CSA, phenotype, and morphology were completed with factorial ANOVA. When needed, ANOVA and Holm-Sidak-corrected post-hoc tests were performed. Non-parametric data are presented as median (interquartile range, IQR), while parametric results are given as mean (±SD). The level of significance was p<0.05.

## Results

Four animals did not complete the experimental protocol due to infection (n = 1,) or occluder failures (n = 3) and were excluded from analysis. Table 1 summarizes the age and body mass of each group of animals. Animals in the ITTO-20 and ITTO-PAR groups were older than ITTO animals (p<0.05). Additionally, ITTO-20 animals weighed more than ITTO animals (p<0.01). However, there were no significant group differences in weight gain.

### Experiment #1: Effect of ITTO on diaphragm regional remodeling

Figure 2A depicts the CSA obtained from the dorsal, medial and ventral costal and the crural regions of the diaphragm. The CSA of the ITTO animals was significantly greater in the medial diaphragm than in the other diaphragm regions (ANOVA, p<0.05). Moreover, the CSA of type IIx/b fibers was significantly larger in the medial and crural regions of ITTO animals, compared to SHAM animals (ANOVA, Holm-Sidek contrasts, p<0.05). Regardless of the region or group assignment, type IIx/b fibers were significantly larger than type IIa or I fibers (ANOVA, p<0.001).

Figures 2B and 2C show the regional fiber proportions and MHC A_A_ of the diaphragm. MHC A_A_ of type IIx/b fibers was significantly lower in the ventral and crural diaphragms of SHAM-conditioned animals (ANOVA, Holm-Sidek contrasts, p<0.01). Connective tissue tended to be more prevalent in the crural region (Table 2), but this difference did not reach statistical significance (p = 0.055). The medial costal diaphragms from ITTO- and SHAM-conditioned animals are shown in Figure 3. Subsequent experiments focused upon the medial costal diaphragm, the region of greatest hypertrophy.

### Experiment #2: Effect of ITTO training duration on diaphragm remodeling

**Figure 4:**
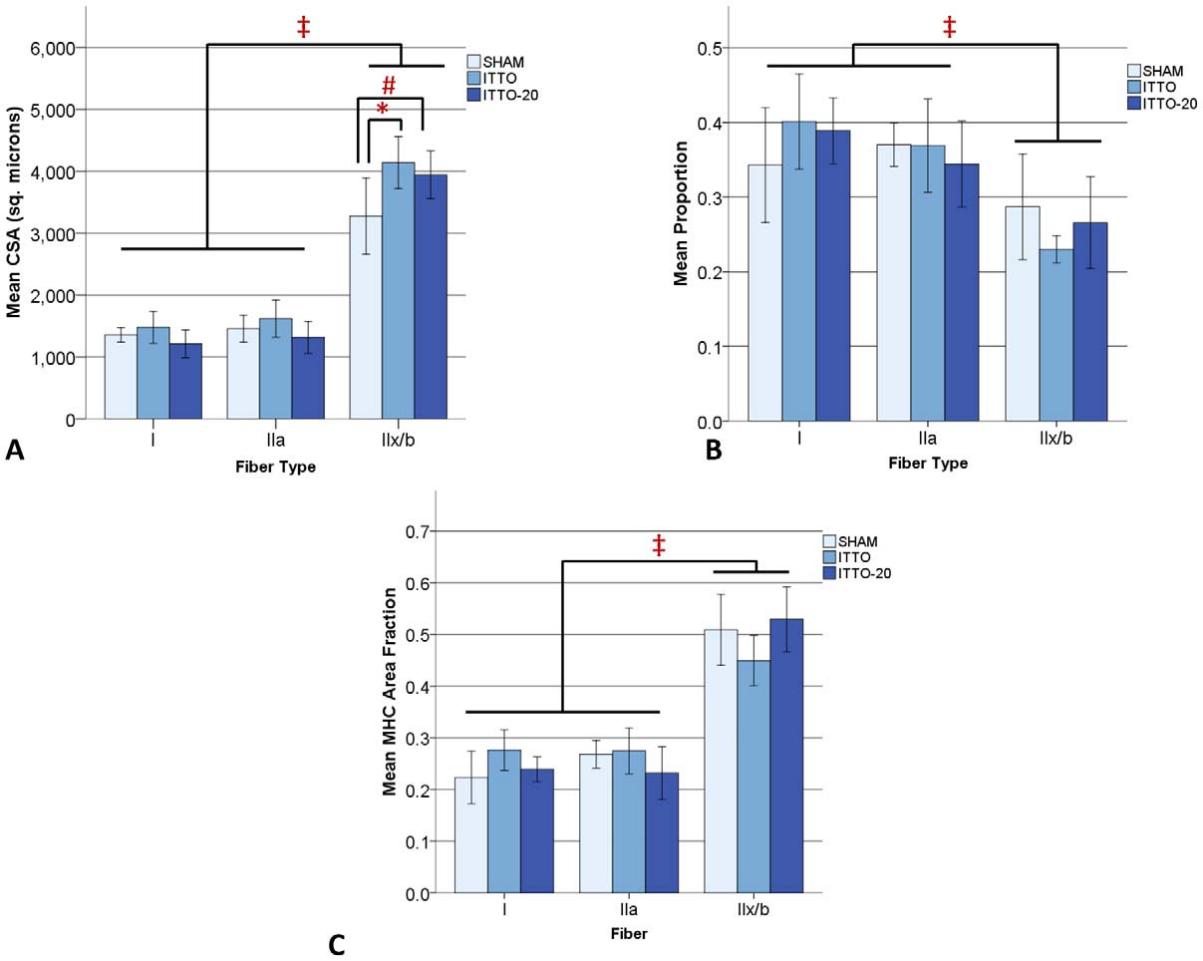
Remodeling of the medial costal diaphragm after 10- or 20-minute ITTO conditioning sessions. (**A**) CSA in type IIx/b fibers was significantly greater for ITTO (# p<0.01) and ITTO-20 (* p<0.05) animals, compared to SHAM animals. In all conditions, type IIx/b fibers were significantly larger than type I or IIa fibers (‡ p<0.001). (**B**) Type IIx/b fibers were significantly less prevalent than type I or type IIa (‡ p<0.001). The duration of ITTO sessions did not influence fiber type proportions. (**C**) Type IIx/b fibers occupied the largest A_A_ (‡ p<0.001), but there were no significant group variations in MHC A_A_.

Figure 4 compares fiber remodeling of the medial costal diaphragm by ITTO, ITTO-20 or SHAM conditioning. Type IIx/b fibers were significantly larger in the ITTO and ITTO-20 groups, when contrasted with SHAM-conditioned animals (ITTO: 4141±419, ITTO-20: 3943±386, SHAM: 3214±581 µm^2^, p<0.005). CSA did not differ between the ITTO and ITTO-20 animals. In all groups, type IIx/b fibers were significantly larger than oxidative fibers (p<0.001).

In every group, type IIx/b fibers were least prevalent (p<0.001) yet occupied a significantly larger fiber type A_A_ (0<0.001). There were no group variations for fiber type proportions or MHC A_A_., but the proportion (p = 0.14) and myosin area fraction (p = 0.10) of type IIx/b fibers trended lower for the ITTO group. Morphological analysis showed a significantly smaller A_A_ of connective tissue in ITTO-20 animals (p<0.005). The relative prevalence of normal muscle and remodeled cells did not differ between groups (Table 3).

**Table 3:** Area fraction (A_A_) of remodeled cells in the medial costal diaphragm after 10-minute occlusion sessions (ITTO), 20-minute occlusion sessions (ITTO-20) or sham conditioning.

	Normal fibers %	Remodeled fibers* %	Connective tissue* %
SHAM	83.3±1.9	4.8±0.9	9.0±0.7
ITTO	82.7±4.5	5.0±2.0	9.5±1.8
ITTO-20	85.5±2.4	3.7±1.6	10.8±2.8

Values are mean ± SD. *p<0.001Significant main effect for category: significantly different A_A_ from normal.

### Experiment #3: Effect of full or partial ITTO on diaphragm remodeling

**Figure 5:**
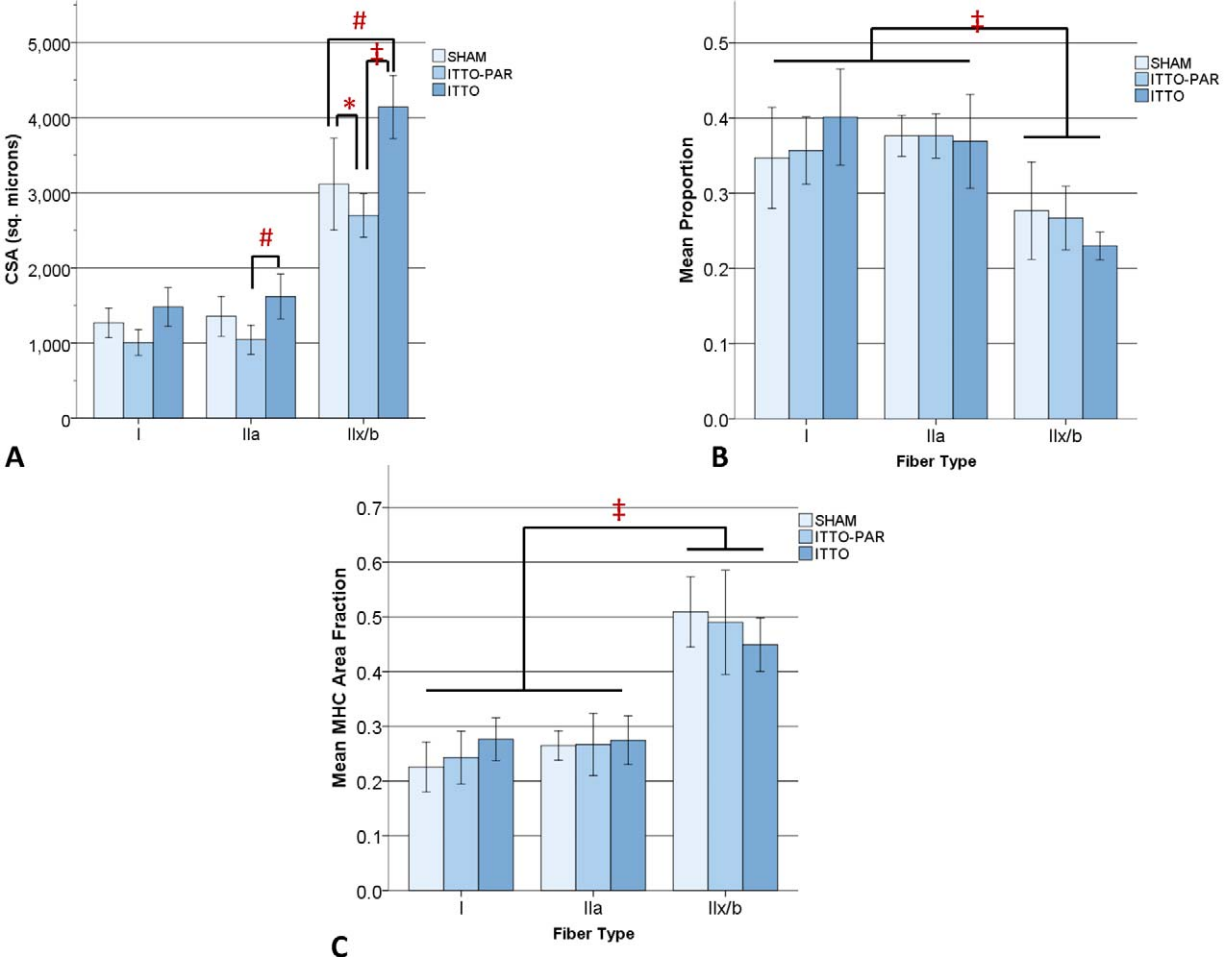
Medial costal diaphragm remodeling after complete or partial ITTO. (**A**) CSA was significantly larger in type IIx/b fibers for ITTO, compared to ITTO-PAR and SHAM animals (‡ p<0.001). In addition, SHAM animals had significantly larger type IIx/b fibers than ITTO-PAR animals (* p<0.05). Type IIa fibers were significantly larger in ITTO animals, compared to ITTO-PAR (# p<0.01). (**B**) Although there were significantly fewer type IIx/b fibers in all groups (‡ p<0.001), training did not affect fiber type proportions. (**C**) Type IIx/b fibers occupied the largest A_A_, regardless of group (‡ p<0.001).

The medial costal diaphragm CSA, fiber proportions and A_A_ of the ITTO, ITTO-PAR and SHAM animals are presented in Figure 5. Type IIx/b fibers were significantly larger in ITTO animals (ITTO: 4141±419, ITTO-PAR: 2698±289, SHAM: 3214±581 µm^2^, p<0.001). Furthermore, type IIx/b fiber CSA was significantly larger in SHAM animals than ITTO-PAR animals (p<0.05). For type IIa fibers, CSA was significantly greater in ITTO animals, compared to ITTO-PAR animals (ITTO: 1598±313, ITTO-PAR: 1045±193 µm^2^, p<0.01).

Regardless of training group, there were significantly fewer type IIx/b fibers, and the A_A_ occupied by type IIx/b MHC was significantly larger than oxidative A_A_ (p<0.001). Neither fiber composition nor morphology was affected by group assignment.

## Discussion

This experiment provides novel information regarding the effects of short duration, high intensity respiratory muscle overload training with ITTO, on diaphragmatic muscle fiber remodeling. The study of this innovative training regime adds new findings to the existing muscle biology literature in two primary areas: (1) ITTO incorporated brief yet reversible occlusive stimuli that to our knowledge have not been previously utilized for respiratory training, and (2) the training was associated with rapid, preferential type IIx/b fiber hypertrophy in the medial costal and crural diaphragm. Although pre-post changes in diaphragmatic fiber remodeling could not be directly measured in the same animals, inclusion of an operated, sham-trained group accounted for the influences of growth, surgery, and daily handling during the experiment. The findings support the postulate that the ITTO regime could serve as an animal model of rehabilitative overload training to induce diaphragmatic fiber hypertrophic remodeling.

The rodent ITTO model of IMST improves upon prior sustained loading paradigms in rodents. Previous studies that applied sustained loading with tracheal banding for days to weeks showed evidence of hypertrophy in slow, oxidative diaphragm fibers [13], [31]. However, sustained loading induced remodeling was accompanied by fiber injury and inflammation and, in some instances, high animal mortality. These undesirable effects could be related to the permanent respiratory load created by banding. Tracheostomies are not feasible for chronic rodent experiments due to tracheal edema and bleeding complications [32]. Later studies employed nasal masks for flow-resistive respiratory muscle endurance training. This regime elicited type IIx/b 9–32% hypertrophy and mid-costal diaphragm fiber CSA enlargement of all fiber types after 8 weeks [33], [34]. Our ITTO model was advantageous over previous loading regimes because it provided short duration, reversible occlusive loads without banding the trachea or implanting a permanent tracheostomy, and it resulted in significant fiber CSA differences after 10 sessions. Further, animals subjected to ITTO gained a similar amount of weight as the SHAM group, which we speculate means the loading regime was well-tolerated. The present animal ITTO model is also advantageous because inspiratory muscle hypertrophy can be studied without widespread morphological remodeling (Table 4) and few complications.

**Table 4:** Area fractions of remodeled tissue, including internally-nucleated cells, small/angular fibers, inflamed/necrotic fibers, and inflammatory cells.

	Internally nucleated fibers %	Small angular fibers %	Inflamed, necrotic fibers %	Cytoplasm changes %	Inflammatory cells %
SHAM	1.0±1.0	0.3±0.4	0.6±0.5	0	1.5±0.6
ITTO	1.0±0.6	0.7±0.6	0.5±0.6	0	1.5±0.8
ITTO-20	1.7±1.2	0.1±0.1	0.9±0.9	0.1±0.0	1.2±0.6
ITTO-PAR	1.4±2.3	0.3±0.4	0.9±0.6	0	2.0±1.0

While existing studies of diaphragm histology frequently sampled from a small portion of the costal region [13], [31], [33]–[35], we extracted tissue from four distinct areas throughout the muscle and found regional differences in remodeling. Medial costal diaphragm fibers hypertrophied to a greater extent than other regions. By way of a sizeable appositional zone and relatively large mass, the medial costal diaphragm may have a kinematic advantage over other costal regions, to facilitate ribcage expansion during escalated respiratory demands [22], [36]. Further, co-activation of the synergist intercostals reduces diaphragmatic shortening [37]–[39] and in some cases lengthens fibers in the mid-costal region during airway occlusion [40]. While lengthening muscular contractions can facilitate cellular disruption, they accelerate rates of hypertrophy in the limb muscles [41]. The regional kinematics and mechanical advantage of the diaphragm during airway occlusion are not fully understood and may be species-dependent.

We also found modest yet significant CSA differences in the crural region that could be related to the additional postural [42] and lower esophageal sphincter [43] functions of the crural diaphragm. Rat motor unit activity appears uniform in the crural and costal segments with inspiration and non-ventilatory behaviors [44], [45], and injurious mechanical loads typically induce greater rat costal than crural diaphragmatic remodeling [21], [30]. However, the phrenic neural distribution may be greater in the crural region [46], and some cuff inflations could have occurred at times other than end-exhalation, resulting in occasional crural motor unit recruitment during the expiratory phase of ventilation [47]. Further study in conscious animals utilizing ITTO is suggested.

ITTO training preferentially remodeled type IIx/b fibers. Recruitment of faster, fatigable diaphragmatic motor units corresponds to the intensity of the mechanical load, and the activation may influence the pattern of hypertrophy [48], [49]. During eupnea, slow, oxidative motor units are predominately active, innervating fatigue-resistant type I muscle fibers. A greater load magnitude is crucial to facilitate preferential type II fiber hypertrophy [50], [51], yet brief, high pressure inspiratory loads have not been widely examined in animal or clinical respiratory training regimes. The load magnitude was not calculated in the current study but an occlusion is an infinite resistive load. Previous research in anesthetized animals and conscious humans indicates that brief airway occlusions elicit robust diaphragmatic activation [52] and high inspiratory pressures [48], [53], [54]. We speculate ITTO also increased activation of the diaphragm [27] and other respiratory pump muscles needed for fast-fiber remodeling.

We did not find training differences in oxidative fiber CSA and did not reach a statistically significant shift in fiber composition with training, yet the study appears adequately powered. Our *a priori* analysis of ITTO and SHAM pilot data indicated that six animals per group would be needed to identify diaphragmatic fast fiber hypertrophy (Cohen's D = 1.58, 1-β  = .80). Post-hoc examination of our dataset indicated 54 animals per group would have been required to detect diaphragm type I hypertrophy, and 55 animals per group needed to detect type IIa hypertrophy. Therefore, we attributed preferential differences in fast-fiber CSA to the ITTO conditioning. The interaction effect of training group-fiber type approached but did not reach significance (p = 0.10) for type IIx/b fibers of the ITTO animals. Fiber CSA is a sensitive early indicator of training, while an oxidative shift in fiber composition occurs later with resistance training [55]. To conclusively investigate ITTO effects of diaphragm fiber composition, we recommend that future experimental designs incorporate more than 10 training sessions over a longer duration.

The findings did not support the hypothesis that increased durations of ITTO sessions (and therefore increased numbers of respiratory efforts) would elicit additional diaphragm fiber hypertrophy. The older age of the ITTO-20 animals must be considered as a potential confound (Table 1). Advanced age has been associated with impaired protein synthesis and delayed myogenic responses after resistance exercise [56], [57]. However, the oldest ITTO-20 animals were in early to middle adulthood, while the ITTO group was in late adolescence [58]. Thus, we do not expect that aging inhibited the training responses of ITTO-20 animals, supported by the lack of significant differences between the ITTO and ITTO-20 groups [59]. While rat body mass and fiber size increase through late adulthood [60], [61], we did not find larger fibers in the older ITTO-20 than ITTO animals. Alternatively, the 20-minute daily regime resulted in more handling of the animals, which one could speculate served as a stressor. Psychological stress in rodents has also been shown to attenuate weight gain [62] and promote catabolic signaling in skeletal muscle [63]. It is not clear whether the additional training time results in functional changes. We cannot rule out a potential interaction between the age of ITTO-20 animals and hypertrophy responses, and feel this may warrant future study.

In contrast to the ITTO and ITTO-20 groups, diaphragmatic fiber CSA did not increase in animals that received partial obstruction. In ITTO-PAR, inspiratory flow through the narrowed airway was essentially a resistive load. Endurance exercise training reduces the fast MHC content in the diaphragm and upper airway muscles, and this could be due to small imposed resistive loads of exercise hyperpnea [64], [65]. Alternatively, typical respiratory compensation for resistive loads in resting animals extends inspiratory time and reduces peak inspiratory flow [66]. In either case, we speculate that ITTO-PAR training failed to generate a sufficient pressure overload to activate hypertrophy signaling pathways. Load intensities greater than 60% of maximum capacity may be needed to significantly upregulate protein synthesis [67]. Research in humans showed a clear load specificity of IMST [68]. In other words, improvements in maximal inspiratory pressure required training with high to near-maximal intensities, while low-resistance, high-flow training increased peak inspiratory flow without strengthening. We further postulate the high-intensity ITTO pressure load was the essential stimulus for the diaphragmatic fiber hypertrophy response of ITTO and ITTO-20 animals.

Increased diaphragmatic fiber CSA has been reported in other types of muscle plasticity, and they could provide an alternative explanation of the findings. For example, an enlarged fiber CSA can result from passive stretching, injury or myopathy [69]–[71]. We did not find evidence of the widespread morphological remodeling characteristic of these other models. Diaphragmatic fiber hypertrophy has also been reported following chemical or mechanical denervation. However, the current protocol did not result in preferential slow fiber hypertrophy followed by progressive type IIx/b fiber atrophy [72], and we did not visualize small or angular fiber morphology, suggestive of denervation. Moreover, the occluder cuff placement proximal to the spinal emergence of the phrenic nerves reduced the likelihood of neural injury.

From a translational perspective, one may ask whether clinical strength training of the respiratory muscles is the most effective way to improve breathing symptoms in patients. In fact, early generation inspiratory muscle training was designed on the rationale that that if a patient can sustain ventilation against a low load for a prolonged period of time (endurance), than it should improve ventilation. However, the results were not consistently positive for improving ventilator weaning [73], [74]. The mixed results may be due to the slow, low flow breathing pattern some patients adopt during prolonged inspiratory resistive training, as a strategy to reduce the imposed load [75]. The resulting hypoventilation is unsustainable.

It has been noted previously that strengthening exercise may be crucial for ventilator weaning [76], because fast-fatigable fiber atrophy is a characteristic of prolonged MV in animals and humans [6], [77]. Moreover, diaphragm fast-fatigable fibers are activated only with the highest imposed loads and expulsive reflexes [48], [78]. Clinically, IMST of patients has been shown to increase strength, reduce dyspnea, and facilitate weaning from MV [7], [79]–[81]. We speculate that fast-fiber hypertrophy could be particularly beneficial to patients with respiratory muscle weakness and cough dysfunction, which are highly predictive of ventilatory failure [82]–[85]. In healthy humans, respiratory muscle training facilitates activation of the respiratory pump and airway dilator motoneurons, reduces load effort perception, and enhances athletic performance [86]–[89].

Still, it is not known if the human diaphragm hypertrophies with training. The extrapolation of results from small mammals to humans must be made cautiously. Moreover, we appreciate the accessory muscles of ventilation play a substantial role in load-compensation responses [79] and did not analyze whether respiratory muscle force was altered by ITTO. To further the translational applications of training and diaphragmatic muscle remodeling, we recommend that future works examine whether our model of ITTO modifies the pressure-generating capacity of the diaphragm and accessory ventilatory muscles.

In conclusion, our findings indicate that short duration ITTO is a useful high-pressure overload respiratory training regime that facilitates type II fiber hypertrophy in the medial costal diaphragm. A maximal occlusive load was essential to induce type IIx/b diaphragmatic fiber remodeling. This innovative model holds promise as an experimental representation of clinical respiratory muscle strength training, in order to examine mechanistic and behavioral adaptations of training. Further investigations are recommended to determine whether ITTO elicits a functional adaptation of the diaphragm.
