# Correction: Chronic Intrinsic Transient Tracheal Occlusion Elicits Diaphragmatic Muscle Fiber Remodeling in Conscious Rodents

**DOI:** 10.1371/annotation/4d84ef28-da1b-4dba-9df6-e8f2ec51cced

**Published:** 2013-05-16

**Authors:** Barbara K. Smith, A. Daniel Martin, Krista Vandenborne, Brittany D. Darragh, Paul W. Davenport

There was an error in the Funding statement.

The correct version of the statement is: Pre-doctoral training support to BKS was provided by National Institutes of Health T32 HD043730 and The Foundation for Physical Therapy. Some supplies were provided by an Howard Hughes Medical Institute HHMI-funded mini-grant to BDD. Publication of this article was funded in part by the University of Florida Open-Access Publishing Fund. The funders had no role in study design, data collection, decision to publish, or preparation of the manuscript. 

